# Cognitive Behavioral Therapy for Cancer‐Related Fatigue: A Comparison Between Patients Treated With Curative Intent and Patients With Advanced Cancer

**DOI:** 10.1002/pon.70282

**Published:** 2025-09-19

**Authors:** Hannah B. van der Pas, Annemarie M. J. Braamse, Gijs Bleijenberg, Hanneke W. M. van Laarhoven, Pythia Nieuwkerk, Harm Westdorp, Lia van Zuylen, Fabiola Müller, Hans Knoop

**Affiliations:** ^1^ Department of Medical Psychology Amsterdam UMC Location University of Amsterdam Amsterdam the Netherlands; ^2^ Cancer Treatment and Quality of Life Cancer Center Amsterdam Amsterdam the Netherlands; ^3^ Radboudumc Department of Medical Psychology Nijmegen the Netherlands; ^4^ Department of Medical Oncology Amsterdam UMC Location University of Amsterdam Amsterdam the Netherlands; ^5^ Radboudumc Department of Medical Oncology Nijmegen the Netherlands; ^6^ Amsterdam Public Health Mental Health Amsterdam the Netherlands; ^7^ Division of Psychology Department of Clinical Neuroscience Karolinska Institutet Stockholm Sweden

**Keywords:** advanced cancer, cancer, cancer survivors, cancer‐related fatigue, cognitive‐behavioral therapy, oncology, palliative care

## Abstract

**Background:**

Cancer‐related fatigue (CRF) is prevalent in patients who have been treated with curative intent and patients with advanced cancer. Cognitive behavioral therapy (CBT) has been shown effective in reducing CRF in both groups.

**Aims:**

To compare both groups with respect to: (1) pre‐treatment levels of fatigue and fatigue‐perpetuating cognitive‐behavioral factors; (2) the magnitude of the effect of CBT on fatigue; and (3) mediators of the treatment response.

**Methods:**

Data of four randomized controlled trials testing the efficacy of CBT for CRF were pooled, three in patients treated with curative intent (*n* = 249), and one in patients with advanced cancer (*n* = 134). Baseline characteristics were compared with ANCOVAs. Moderation analysis was used to investigate whether the treatment effect differed between groups. With moderated mediation analyses differences in the mechanisms by which CBT reduces fatigue were evaluated.

**Results:**

The two groups differed significantly at baseline on fatigue‐perpetuating factors, but not on fatigue severity. Patients with advanced cancer reported a smaller decrease in fatigue severity following CBT than patients treated with curative intent (*p* = 0.022). The multivariate moderated mediation analysis showed a larger decrease in fatigue catastrophizing in patients treated with curative intent than in patients with advanced cancer.

**Conclusion:**

CBT for CRF has less effect on catastrophizing, a known fatigue‐perpetuating factor and mediator of the effect of CBT, and fatigue severity in patients with advanced cancer. Further research has to determine if the effectiveness of CBT for CRF in advanced cancer patients can be improved.

## 1 Background

Cancer‐related fatigue (CRF) is a prevalent and disturbing symptom for patients throughout the cancer trajectory [1]. In cancer survivors, approximately one quarter of patients continue to experience CRF after completion of cancer treatment [2]. In patients with advanced cancer the prevalence of CRF is estimated to be around 60% [3]. Regardless of the disease stage, CRF has a negative impact on both quality of life and patients' functional status [4].

Cognitive behavioral therapy (CBT) can significantly reduce CRF [5, 6]. CBT for fatigue focuses on identifying and changing unhelpful fatigue‐related thoughts and behaviors, and was specifically developed to target chronic, severe fatigue [7]. The cognitive behavioral model distinguishes between factors that precipitate fatigue and factors that perpetuate fatigue [8]. While cancer and its treatment can trigger CRF, cognitive‐behavioral factors are hypothesized to contribute to the persistence of the fatigue [9]. Well‐known fatigue perpetuating factors are sleep problems, dysregulation of activity or the tendency to catastrophize in response to fatigue [10]. CBT for CRF addresses the fatigue‐perpetuating factors aiming for a reduction of fatigue and associated disability. Factors that have been shown to mediate the effect of CBT on fatigue are changes in fatigue‐related beliefs [9] and reduction in depressive symptoms [9, 11].

CBT for CRF was first developed for and tested in cancer patients treated with curative intent (Gielissen et al. [12]; Prinsen et al. [13]; Abrahams et al. [4]). More recently, CBT has been adapted for patients with advanced cancer (Poort et al. [1]). However, patients treated with curative intent and patients with advanced cancer have never been directly compared with respect to the magnitude of the positive effect of CBT on fatigue or mediators of the treatment response. It seems likely that fatigue is more severe in patients with advanced cancer, as they still have cancer and often receive treatment that triggers symptoms. Additionally, it can be hypothesized that the uncertainty regarding their prognosis may contribute to higher levels of fatigue catastrophizing and/or fear of disease progression, both known to perpetuate CRF [14]. Further, the direct adverse effects of cancer treatment may result in lower activity levels or more sleep problems in patients with advanced cancer, which are also known fatigue‐perpetuating factors [15, 16]. We currently lack insight into the differences in fatigue severity and fatigue‐perpetuating factors between patients that have been treated with curative intent and patients with advanced cancer. It is also unclear if the same cognitive‐behavioral variables mediate the treatment response to the same extent in both groups.

In this study we compared both groups with respect to: (1) pre‐treatment levels of fatigue and fatigue‐perpetuating cognitive‐behavioral factors; (2) the magnitude of the effect of CBT on fatigue; and (3) mediators of the treatment response. Results of these comparisons may prove useful in the further development of CBT for CRF by adapting it to the specific characteristics of the different patient groups, which may enhance the effectiveness of the intervention.

## 2 Methods

### 2.1 Study Design

Data of four randomized controlled trials (RCTs) testing the efficacy of CBT for CRF were pooled and re‐analyzed. Three RCTs evaluated the efficacy of CBT on fatigue severity in patients who have been treated with curative intent. In the trial by Gielissen and colleagues [12], patients were randomly assigned 1:1 to CBT or a waitlist control. In the trial by Prinsen and colleagues [13] patients were assigned 3:1 to either CBT or the waitlist control group. Abrahams' study [4] compared internet‐based CBT (I‐CBT) to care as usual in severely fatigued breast cancer survivors, with a 1:1 ratio. Treatment duration was 6 months in these three studies. All patients were assessed at baseline (T0) and 6 months later (post‐CBT or post‐control condition (T1)). One RCT evaluated the efficacy of CBT for CRF in patients with advanced cancer: Poort and colleagues [1] compared CBT and graded exercise therapy (GET) with usual care for CRF, randomizing patients 1:1:1. The T1 assessment was conducted at 14 weeks, as the CBT for patients with advanced cancer had a duration of 12 weeks. This shorter duration for the advanced cancer patient group was chosen due to their limited life expectancy. The primary outcome of all trials was fatigue severity assessed with the same questionnaire. In all four RCTs, fatigue severity reduced significantly after CBT compared to the control conditions groups. Table 1 provides an overview of the studies, number of patients and the trial arms.

**TABLE 1:** Overview of studies, patient group and conditions.

Study	Patient group	Conditions
Gielissen et al. [12]	Patients treated with curative intent (*n* = 98)	CBT (*n* = 50) Waitlist control (*n* = 48)
Prinsen et al. [13]	Patients treated with curative intent (*n* = 64)	CBT (*n* = 50) Waitlist control (*n* = 14)
Abrahams et al. [4]	Patients treated with curative intent (*n* = 132)	Internet‐based CBT (*n* = 66) Care as usual (*n* = 66)
Poort et al. [1]	Patients with advanced cancer (*n* = 134)	CBT (*n* = 46) GET (*n* = 42) Care as usual (*n* = 46)

*Note:* Sample sizes (*n*) refer to number of participants that were randomized to either the Cognitive Behavioral Therapy (CBT) intervention or the control group, or Graded Exercise Therapy (GET) in the trial of Poort et al. [1].

### 2.2 Participants

Across trials, patients were eligible if they were severely fatigued (score of ≥ 35 on the Checklist Individual Strength, subscale fatigue severity [CIS‐fatigue]) and aged ≥ 18 years. Patients had completed curative treatment of a malignant, solid tumor or of non‐Hodgkin's lymphoma at least 1 year ago in the Gielissen and Prinsen trial [12, 13], or completed treatment for breast cancer at least 3 months previously in the Abrahams trial [4]. Patients in the Poort trial [1] received systemic treatment with palliative intent for a solid tumor and had a life expectancy according to their oncologist of at least 6 months. Since the Abrahams trial investigated internet‐based CBT, internet access was also required in this study. Patients who underwent psychological or psychiatric treatment (e.g., for depression) or had other comorbidities that could explain the presence of their fatigue were excluded. See Supporting Information S1: Table S1 for the main in‐ and exclusion criteria of the studies.

### 2.3 Procedure

After providing written informed consent, patients were enrolled and randomly assigned to CBT or the other condition(s). All RCTs were reviewed and approved by the ethics committees of the Radboudumc and the other participating hospitals. Trial registration numbers are ISRCTN: 44562532 [12], NCT01096641 [13], NTR4309 [4] and NTR3812 [1].

### 2.4 Cognitive Behavioral Therapy/Intervention

CBT for CRF was based on the cognitive‐behavioral model of CRF and consisted of six modules, each addressing a fatigue‐perpetuating factor. Only relevant modules were delivered, determined at baseline by an intake interview and questionnaires assessing the fatigue‐perpetuating factors. The six CBT modules addressed: (1) insufficient coping with cancer and its treatment, (2) unhelpful beliefs regarding fatigue, (3) sleep problems, (4) uneven or low levels of (physical) activity with all patients following a graded activity program, (5) lack of social support, and (6) fear of disease recurrence in curatively treated patients and fear of disease progression in patients with advanced cancer. See Table 2 for an overview of the CBT modules, fatigue‐perpetuating factors and corresponding instruments.

**TABLE 2:** Overview of the CBT modules, fatigue‐perpetuating factors and corresponding instruments.

	CBT module	Fatigue‐perpetuating factor	Instrument
1	Coping	Insufficient coping with cancer and treatment	Impact of event scale (IES) ‐subscale intrusion and subscale avoidance [17]
2	Cognition	Dysfunctional cognitions regarding fatigue: Focusing on symptoms Catastrophizing in response to fatigue Fatigue‐related self‐efficacy	Illness management questionnaire (IMQ‐factor III) [18] Fatigue catastrophizing scale (FCS) [19, 20] Self‐efficacy scale 28 (SES28‐fatigue) [19]
3	Sleep	Dysregulation of sleep	Sickness impact profile (SIP)—Sleep rest subscale [1, 21]
4	Activity	Dysregulation of activity	Checklist for individual strength (CIS)—subscale activity (CIS‐a) [19] Actigraph [19]
5	Social support	Low social support/negative social interactions	Social support list (SSL)—subscales discrepancies, interactions and negative interaction [18]
6	Fear of recurrence^a^	Fear of disease recurrence	(Modified) cancer acceptance scale (CAS) [22]

^a^ Fear of recurrence was only measured in the studies with patients treated with curative intent.

### 2.5 Control Conditions

In the RCTs of Gielissen et al., Prinsen et al. and Abrahams et al. patients not assigned to CBT were placed on a waiting list. Patients in the waitlist control group could receive CBT after the end of the study. In the Poort trial both CBT and graded exercise therapy (GET) were compared to care as usual. Patients randomized to GET received a 12‐week supervised exercise program. Data of all patients were used for the comparison of the baseline values of cognitive‐behavioral perpetuating factors and fatigue. For the comparison of treatment effect and mediators, only data from the CBT and care as usual condition of the Poort trial were used.

### 2.6 Outcome Measures

Participants self‐reported demographic characteristics at baseline. Clinical variables were derived from medical records or were self‐reported. In the latter case, information was verified with their general practitioner or the treating consultant.

### 2.7 Primary Outcome

Fatigue severity was measured using the subscale fatigue severity (8 items, 7‐point Likert scale) of the Checklist Individual Strength (CIS‐fatigue). Scores range from 8–56. A score of 35 points or higher is the validated cut‐off score for severe fatigue [23]. The CIS‐fatigue has been used in multiple intervention studies and proved to be sensitive to change. It has good reliability (Cronbach's alpha = 0.88) and discriminative validity [23].

### 2.8 Fatigue‐Perpetuating Factors and Putative Mediators

Fatigue‐perpetuating factors were: problems with coping with cancer, focusing on symptoms, fatigue catastrophizing, low self‐efficacy with respect to fatigue, sleep problems, self‐reported problems with activity, objective physical activity measured by actigraphy, and perceived lack of social support. In addition, depressive symptoms were assessed as a putative mediator. Fear of cancer recurrence was measured in the patients treated with curative intent. For patients with advanced cancer, a fear of progression module was used instead, precluding direct comparisons. Therefore, anxiety was assessed as a proxy measure for fear of recurrence/progression. See Table 2 for fatigue‐perpetuating factors and corresponding instruments. See supplementary materials for details of all instruments.

### 2.9 Statistical Analyses

All statistical analyses were conducted in SPSS (version 28.0.1.1). The variables age and sex were included as covariates in all analyses.

Research aim 1: comparison of fatigue severity and levels of fatigue‐perpetuating factors between both groups before CBT‐intervention.

Differences in baseline scores in fatigue and fatigue‐perpetuating factors between both groups were investigated using analysis of covariance (ANCOVA). Univariate analyses were conducted with patient group (curative intent vs. advanced cancer) as the independent variable and fatigue severity and fatigue‐perpetuating factors at T0 as dependent variables. These analyses were conducted with a Bonferroni adjustment. Partial eta squared (ηp^2^) was calculated as an effect size index: ηp^2^ = 0.01 indicates a small, ηp^2^ = 0.06 a medium, and ηp^2^ = 0.14 a large between‐group difference [24].

Research aim 2: comparison of the magnitude of the effect of CBT on fatigue severity between both patient groups.

To determine whether patient group moderates the effect of condition (CBT vs. control) on changes in CRF a moderation analysis was performed using the PROCESS macro (version v4.2 by Andrew F Hayes) in SPSS; see figure S1, supplementary materials. The residual change score of fatigue severity was used, accounting for the baseline value (fatigue severity at T0). This approach allowed for the determination of the change in fatigue severity, independent of the influence from fatigue severity at T0 [25].

Research aim 3: to investigate differences in working mechanisms (fatigue‐perpetuating factors) explaining the CBT effect between patient groups.

First, multiple mediation analyses were conducted with single putative mediators (univariate). In these models, condition was the independent variable. The residual change score of the fatigue‐perpetuating factors (only including factors measured both pre‐ and post‐intervention) was entered as a potential mediator, and the residual change score of fatigue severity was entered as dependent variable.

After the univariate mediation analyses, patient group was included as a putative moderator of the a‐path, b‐path and c’‐path (see figure S2 in the supplementary materials). This step was applied across all fatigue‐perpetuating factors measured post‐intervention, regardless of the significance in the univariate mediation models. These univariate moderated mediation analyses were performed to explore whether the working mechanisms differed per patient group. The index of moderated mediation was used to determine if the mediation effect (a‐path * b‐path) varied between the two groups.

Finally, a multivariate moderated mediation analysis was conducted in which only those fatigue‐perpetuating factors were included for which we found a significant mediation effect in the univariate moderated mediation analysis. In this analysis, the condition (CBT vs. control) was entered as independent variable, the residual change score in fatigue severity as outcome, the residual change scores of fatigue‐perpetuating factors as putative mediators, and patient group as moderator.

### 2.10 Sensitivity Analyses

The T1 assessment in the Poort trial was conducted at 14 weeks, which was a shorter period of time compared to the T1 assessments in the other RCTs (6 months). In the Poort trial, a follow‐up measurement was performed after 18 weeks (T2) and again at approximately 6 months (T3) after randomization. A sensitivity analysis was conducted to compare the different time points. The residual change score from T0 to T1 from the RCTs of patients treated with curative intent were compared to the residual change score of T0 to T3 of patients with advanced cancer, as both were conducted at approximately 6 months. These analyses were performed for both our moderation analysis (research aim 2) and our multivariate moderated mediation analysis (research aim 3).

## 3 Results

The demographics and clinical characteristics are presented in Table 3. A significant difference in age and sex was found between the two groups. Therefore, both variables were included as covariates in the analyses.

**TABLE 3:** Demographic and clinical characteristics at baseline.

Characteristics	Patient group		*p*‐value
	Patients treated with curative intent (*n* = 294)	Advanced cancer patients (*n* = 134)	
Age in years: Mean (SD)	48.5 (9.49)	62.8 (9.35)	< 0.001
Sex: Female: *n* (%)	212 (72.1)	77 (57.5)	0.004
Cancer type: *n* (%)			
Breast	185 (62.9)	54 (40.3)	
Colorectal	4 (1.4)	34 (25.4)	
Prostate	3 (1)	27 (20.1)	
Testicular	32 (10.9)	0 (0)	
Gynecological	10 (3.4)	6 (4.5)	
Other	57 (19.4)	13 (9.7)	
Missing	3 (1)	0 (0)	
Time since cancer treatment, in months, mean (SD)	46.7 (43.6)	Not applicable	
Time since first cancer diagnosis, in months, mean (SD)	Not applicable	70.0 (63.3)	
Previous treatment: *n* (%)		Not applicable	
Surgery only	30 (10.2)		
Surgery and radiotherapy	33 (11.2)		
Surgery and chemotherapy	79 (26.9)		
Surgery, radiotherapy and chemotherapy	128 (43.5)		
No surgery, only radiotherapy and/or chemotherapy	24 (8.2)		
Current treatment: *n* (%)	Not applicable		
Chemotherapy		39 (29)	
Hormonal therapy		37 (28)	
Targeted therapy		15 (11)	
Chemotherapy + targeted therapy		27 (20)	
Hormonal therapy + targeted therapy		7 (5)	
Immunotherapy		5 (4)	
Missing		4 (3)	

*Note:* SD = standard deviation.

### 3.1 Comparison of Fatigue Severity and Levels of Fatigue‐Perpetuating Factors Before CBT‐Intervention

**FIGURE 1:**
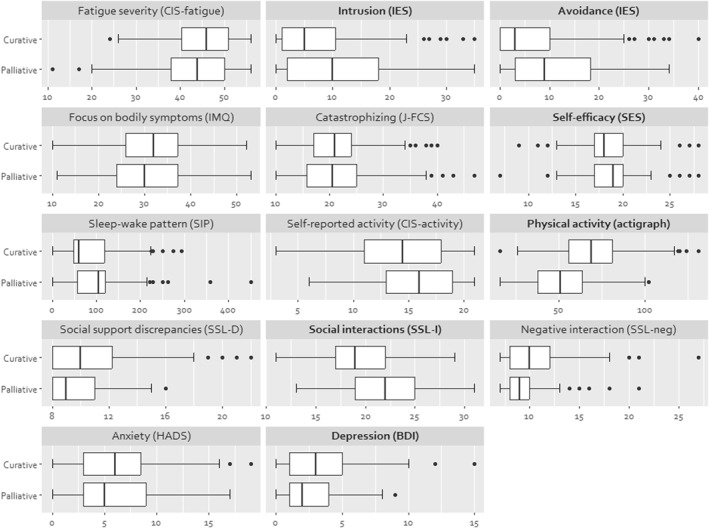
Cancer‐related fatigue (CRF), fatigue‐perpetuating factors and putative mediators at baseline. Baseline data of patients randomized to Graded Exercise Therapy (GET) are included in these analyses. Statistically significant group differences (*p* < 0.05) are printed in bold. Based on estimated marginal means; adjusted outcome means after controlling for age and sex. Higher scores on the variables self‐efficacy, physical activity (actigraph) and social support indicates a positive/better outcome. For all other outcomes, a higher score indicates a negative/poorer outcome.

No significant difference was found in fatigue severity between the two patient groups at baseline. Besides considerable overlap, the following fatigue‐perpetuating factors differed statistically significant between groups at baseline (see Figure 1): patients treated with curative intent had significantly lower scores on the impact of events scale, both on the intrusion (ηp^2^ = 0.058) and avoidance (ηp^2^ = 0.033) subscale and had a higher level of physical activity measured by actigraphy (ηp^2^ = 0.065) as compared to patients with advanced cancer. Patients treated with advanced cancer reported a significantly higher level of self‐efficacy with respect to fatigue (ηp^2^ = 0.014), experienced more social support (ηp^2^ = 0.066), and had less depressive symptoms (ηp^2^ = 0.015) than the curatively treated group.

### 3.2 Comparison of the Magnitude of the Effect of CBT on Fatigue Severity

Our moderation analysis showed a significant interaction of condition and patient group (*p* = 0.022). The reduction in fatigue severity was larger following CBT in patients treated with curative intent (*B* = −1.05, *t* = −9.63, *p* < 0.001) as compared to patients with advanced cancer (*B* = −0.54, *t* = −2.86, *p* = 0.005). As post hoc analysis, we conducted the moderation analysis including all baseline variables that were significantly different between groups at baseline, as covariates (added sequentially). The difference in CBT effect on fatigue was no longer significant after entering depressive symptoms and self‐efficacy as covariates. Supporting Information S1: Table S2 shows the results of the moderation analysis; Supporting Information S1: Tables S3 and S4 present the results of the post hoc moderation analyses.

### 3.3 Differences in Working Mechanisms (Fatigue‐Perpetuating Factors) Explaining the CBT Effect Between Patient Groups

Improvements in self‐reported activity, catastrophizing, self‐efficacy and sleep significantly mediated the effect of CBT on fatigue severity. Change in activity measured by actigraphy was no significant mediator. The univariate moderated mediation models showed an interaction of condition and patient group predicting changes in two putative mediators (a‐paths). In curatively treated patients, CBT resulted in a more pronounced decline in catastrophizing (a‐path, *B* = 0.91, *p* > 0.001) and sleep problems (a‐path, *B* = 0.51, *p* = 0.03) compared to patient with advanced cancer. None of the b‐paths were significantly moderated. The index of moderated mediation, indicating whether the mediation effect (a‐path * b‐path [ab]) is significantly different between patient groups, was only significant for catastrophizing (ab = 0.48, 95% CI (0.23, 0.75)). This indicates that CBT reduced fatigue severity via catastrophizing in curatively treated patients whereas this mechanism was not statistically significant in patients with advanced cancer.

**FIGURE 2:**
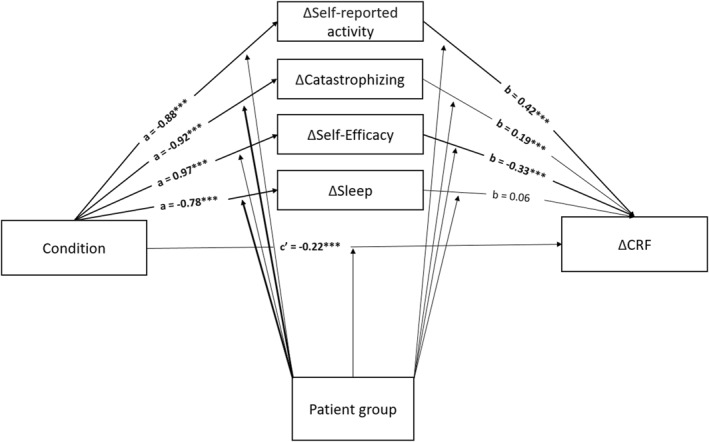
Multivariate moderated mediation analysis. *p < 0.05, **p < 0.01, ***p < 0.001. ∆ = residual change score.

In the multivariate moderated mediation analysis (see Figure 2), changes in self‐reported activity, catastrophizing and self‐efficacy mediated the effect of CBT on fatigue severity. The index of moderated mediation indicates that these relationships do not differ between patients treated with curative intent and patients with advanced cancer for self‐reported activity and self‐efficacy. For catastrophizing, this mediation effect (a‐path * b‐path [ab]) was significant for both groups, but differed significantly between the groups (ab = 0.18, 95% CI (0.06, 0.33)). The mediation effect through catastrophizing was smaller in patients with advanced cancer than cancer survivors. Furthermore, the a‐path from CBT to sleep was significantly moderated by patient group but sleep was no longer a mediator in the multivariate model. The c’‐pathway showed that CBT had a significant direct effect on changes in fatigue severity when controlling for the mediators (*B* = −0.22, *p* = 0.04).

Our sensitivity analysis (multivariate moderated mediation analysis including residual change scores of the variables between T0 and T3, instead of T0 to T1 in the Poort trial) did not lead to different conclusions. The results are illustrated in Supporting Information S1: figure S3.

## 4 Discussion

Pooled data from four randomized controlled trials (RCTs) were analyzed to examine differences between patients treated with curative intent versus patients with advanced cancer in fatigue severity, fatigue‐perpetuating factors, and the magnitude of the response to CBT. We found no differences in fatigue severity between patient groups at baseline which may be due to a limited variance in fatigue, given that patients were selected for the trials based on presence of severe fatigue. Although there was considerable overlap in fatigue‐perpetuating factors, we found that patients treated with curative intent showed significantly fewer intrusions and less avoidance of thinking about cancer and cancer treatment and a higher objectively measured physical activity level at baseline compared to patients with advanced cancer. The Impact of Event Scale measures the traumatic nature of the events surrounding cancer and its treatment. It is perhaps not surprising that patients with advanced cancer score higher on this scale due to the ongoing psychological distress from physical symptoms caused by cancer and its treatment, functional decline and uncertainty about life expectancy [26, 27]. With respect to physical activity, literature comparing patients receiving palliative care to healthy individuals shows that patient report more sedentary behavior, probably due to the burden of ongoing treatment and the symptoms of cancer [28]. To our knowledge, this study is the first to directly compare physical activity levels between patients with advanced cancer and patients who have been treated with curative intent. Interestingly, patients with advanced cancer had significantly higher self‐efficacy, experienced more social support, and fewer depressive symptoms compared to the curatively treated group at baseline. Literature indicates that fatigued cancer survivors often feel less supported with respect to the management of their fatigue after curative treatment, as it is generally expected that their well‐being would improve [29], despite ongoing cancer‐related symptoms [29, 30].

We hypothesized that the effect of CBT on fatigue would be larger in the curatively treated group compared to the advanced cancer patients group, supported by the higher percentages of clinically relevant improvement in fatigue severity in cancer survivors (73%) [4], compared to patients with advanced cancer (63%) [1]. This hypothesis was confirmed. However, post hoc analysis showed that, after controlling for baseline scores on self‐efficacy and depressive symptoms in the moderation model, the interaction effect was no longer significant. Changes in fatigue‐related beliefs and reduction of depressive symptoms have been shown to mediate the effect of CBT on fatigue [9, 11]. Although CBT for fatigue does not directly target depressive symptoms, it addresses emotional distress. The results from the post hoc analysis suggest that the difference in the magnitude of effect of CBT between group may be explained by the fact that the palliative group scores more favorably on depressive symptoms and self‐efficacy and hence might profit less from CBT which influences both aspects.

Our multivariate moderated mediation analysis revealed that an increased self‐reported activity, reduced tendency to catastrophize and increased self‐efficacy were significant mediators of the CBT effect, across patients treated with curative intent and patient with advanced cancer. Although sleep was no longer identified as a mediator in the multivariate model, previous research showed that CBT for insomnia significantly reduces CRF, largely via improved sleep. This suggests that addressing sleep problems is an important component of CBT for fatigue, especially in those patients who report sleep problems [31, 32]. That physical activity, measured by actigraphy, is not a significant mediator of the effect of CBT on fatigue is in line with previous findings [33]. There was a significant difference between patient groups in the mediation effect of catastrophizing. For patients with advanced cancer the mediation through catastrophizing was smaller in size compared to the effect in patients treated with curative intent. The shorter treatment duration could partially explain the reduced effect, perhaps more time is needed to address the tendency to catastrophize in response to fatigue. Also, fatigue catastrophizing may be more challenging to treat in patients with advanced cancer than in patients who have been treated with curative intent as they are confronted with ongoing and life threatening illness. Patients with advanced cancer often face persistent physical symptoms, functional decline and have less future prospects [34], which may reinforce negative beliefs about fatigue and its impact [35]. Catastrophizing may be more resistant to change in patients with advanced cancer due to the persistent existential threat, which can constrain cognitive flexibility and hopefulness. This could limit the effectiveness of traditional cognitive restructuring approaches. Perhaps third‐wave cognitive‐behavioral approaches, emphasizing process‐based change strategies like cognitive defusion and acceptance can help to address the tendency to catastrophize in response to fatigue [36].

Our mediation analysis showed that CBT had a significant direct effect on changes in fatigue severity even after controlling for the mediators suggesting that changes in other (fatigue‐perpetuating) factors also contribute to the observed reduction in fatigue. Previous mediation analyses in other conditions revealed that the effects of CBT on fatigue are achieved through similar mechanisms as found in the current study [37, 38, 39]. An increased self‐efficacy, self‐reported activity, change in beliefs about the ability to be active and a reduction in catastrophizing are repeatedly identified as mediators of the effect of CBT. These findings fit with the idea that chronic fatigue is a transdiagnostic symptom which can be addressed in a similar way across conditions by addressing transdiagnostic fatigue‐perpetuating factors.

### 4.1 Study Strengths

This study is the first to compare fatigue severity, fatigue‐perpetuating factors, and the magnitude of the effect of CBT and its mechanisms in patients with advanced cancer versus patients following curative treatment. Another strength of this study is the use of (nearly) identical inclusion criteria, instruments to assess mediators and outcomes and interventions across studies, which allowed for meaningful comparisons to be made.

### 4.2 Study Limitations

This study is an additional analysis of data collected in studies not specifically powered for the current research questions. We might have missed some differences due to limited power. Relatedly, in the Poort trial only a few variables were measured at follow‐up, which prevented us from analyzing some putative fatigue‐perpetuating factors. Furthermore, since fatigue‐perpetuating factors and cancer‐related fatigue were measured at the same time, we cannot draw definite conclusions regarding the direction of their relationship [9].

Another relevant limitation of this study arises from the difference in the duration of CBT and follow‐up between the groups. While curatively treated patients received CBT‐treatment over a 6‐month period, patients with advanced cancer received a shortened intervention of 12 weeks, which was chosen due to their limited life expectancy. Literature shows that there is a large variation in the time needed to reduce fatigue to normal levels using CBT. A study with patients with chronic fatigue syndrome showed that a quarter of the patients were less severely fatigued within 6 weeks, whereas others remained severely fatigued after 18 weeks of CBT [40]. Both the discrepancy in the duration of CBT and the time to follow‐up could have influenced the observed outcomes. Nevertheless, our sensitivity analyses suggested that our results seem not to have been biased through difference in time interval to follow‐up. Further research on extending the intervention or more specifically addressing catastrophizing beliefs, perhaps by applying alternative strategies such as cognitive defusion and acceptance instead of traditional cognitive restructuring approaches can help to enhance the effectiveness of CBT for patients with advanced cancer.

### 4.3 Clinical Implications

Since the patients with advanced cancer received CBT for only 12 weeks, it may be considered to extend the treatment duration for patients in the palliative phase. Exploring the use of alternative interventions focused on reducing the tendency to catastrophize in response to fatigue may further decrease fatigue in the latter patient group.

## 5 Conclusion

Differences in some fatigue‐perpetuating factors exist between patients treated with curative intent and patients with advanced cancer prior to CBT. The effect of CBT for cancer‐related fatigue is larger in patients having received curative treatment than in patients with advanced cancer. This may however be related to differences in baseline characteristics related to the effect of CBT. An increased level of self‐reported activity, a reduction in the tendency to catastrophize in response to fatigue and an increased self‐efficacy with respect to fatigue mediate the reduction of fatigue in both patient groups. However, the effect of CBT on catastrophizing is weaker in patients with advanced cancer. Extending the duration of CBT and/or placing greater emphasis on reducing catastrophizing in the palliative group, may help improve the effectiveness of CBT in this patient group.

## Author Contributions

**Hannah B. van der Pas:** conceptualization, data curation, formal analysis, investigation, methodology, writing – original draft preparation. **Annemarie M. J. Braamse:** conceptualization, writing – review and editing. **Hanneke W. M. van Laarhoven:** writing – review and editing. **Gijs Bleijenberg:** writing – review and editing. **Pythia Nieuwkerk:** formal analysis, investigation, methodology, writing – review and editing. **Harm Westdorp:** writing – review and editing. **Lia van Zuylen:** writing – review and editing. **Fabiola Müller:** conceptualization, data curation, methodology, supervision, writing – review and editing. **Hans Knoop:** conceptualization, funding acquisition, acquisition of data, investigation, methodology, project administration, resources, supervision, writing – review and editing.

## Conflicts of Interest

The authors declare no conflicts of interest.

## Supporting information


Supporting Information S1

## Data Availability

The data that support the findings of this study are derived from four randomized controlled trials, and are available upon reasonable request. All principal investigators of the original studies have contributed to the manuscript and have agreed to the use of the pooled data for this research. Access may be subject to approval by the principal investigators to ensure compliance with ethical guidelines and participant confidentiality.
