# Supplementary material for: Cognitive Behavioral Therapy for Cancer‐Related Fatigue: A Comparison Between Patients Treated With Curative Intent and Patients With Advanced Cancer
Source: Psychooncology. 2025 Sep 19;34(9):e70282. doi: 10.1002/pon.70282 (PMC12449572; doi:10.1002/pon.70282)
Supplement: Supplementary file 1 — Supporting Information S1 [file PON-34-e70282-s001.docx]

**Supplementary materials**

**Table S1.** Main in-and exclusion criteria of all four randomized controlled trials.

|  | Gielissen et al. | Prinsen et al. | Abrahams et al. | Poort et al. |
| --- | --- | --- | --- | --- |
| **Inclusion criteria:** |  |  |  |  |
| Age | 18- 65 years | 19-65 years | ≥18 years | ≥18 years |
| Diagnosis |  | Malignant, solid tumor | Breast cancer | Incurable cancer, solid tumor |
| Fatigue severity | CIS-fatigue ≥35 | CIS-fatigue ≥35 | CIS-fatigue ≥35 | CIS-fatigue ≥35 |
| Treatment | Completion of treatment for cancer minimal 1 year ago | Completion of treatment for cancer minimal 1 year ago | Completion of treatment for cancer minimal 3 months ago | Systemic treatment with palliative intent |
| Disease stage | Disease free at entry of the study | Disease free at entry of the study | Disease free at entry of the study. | N.A. |
| **Exclusion criteria:** |  |  |  |  |
| Alternative explanation for presence of fatigue | Somatic comorbidity that explain the presence of fatigue | Somatic comorbidity that explain the presence of fatigue | Somatic comorbidity that explain the presence of fatigue | Somatic comorbidity that explain the presence of fatigue |
| Current treatment | Current psychology or psychiatric treatment | Current psychology or psychiatric treatment | Current psychology or psychiatric treatment | Current psychology or psychiatric treatment |
| Brain tumor | Brain tumor in the past |  |  | Symptomatic brain metastases |

*Note: CIS = checklist individual strength.*

**Description of instruments used to assess the putative mediators**

***Problems coping with cancer and cancer treatment*** was assessed using the impact of event scale (IES) (1, 2). Fifteen items (e.g. ‘I tried to get it out of my memory’) assess patients’ intrusive cognitions and avoidance of reminders about cancer and its treatment during the past two weeks. Items are scored on a 4-point scale (scoring: 0-1-3-5) from (0) ‘Not at all’ to (5) ‘often’. 7 items measured intrusion, 8 items measured avoidance. Higher scores indicates more intrusive cognitions or avoidance behavior. The impact of event scale was only measured at baseline for the Poort trial.

***Focusing on fatigue*** was assessed with Illness Management Questionnaire (IMQ) (3). Nine items (e.g. ‘I spend a lot of time thinking about my fatigue’) assess patients’ focus on fatigue during the previous month. Items are scored on a 6-point Likert scale, ranging from (1) ‘Never’ to (6) ‘Always’. Higher scores indicated more focusing on fatigue (range 9-54). Focusing on fatigue was not assessed in the Gielissen-trial and only measured at baseline for the Poort trial.

***Fatigue catastrophizing*** was assessed with the Fatigue Catastrophizing Scale (J-FCS) (4). Ten items (e.g. ‘I imagine the fatigue becoming even more intense and exhausting’) assess patients’ negative cognition and feelings of helplessness about fatigue. Items are scored on a 5-point Likert scale from (1) ‘Never true’ to (5) ‘All the time’. Higher scores indicate more catastrophizing (range 0-52). This was measured in the Abrahams, Prinsen and Poort trial.

***Self-efficacy*** was assessed with the self-efficacy scale (SES-28) as used in previous studies (5). Seven items (e.g. ‘I think I could positively influence my fatigue’) assess patients’ perceived control over their fatigue. Items are scored on a 4-point Likert scale ranging from (1) ‘No, I am convinced that is not true’ to (4) ‘Yes, I am convinced that is true’. Higher scores indicated higher self-efficacy regarding fatigue.

***Sleep disturbance*** was assessed with the subscale sleep and rest of the Sickness Impact Profile-8 (SIP8) (6). Seven items (e.g. ‘I spend much of the day lying down to rest’) assess patients’ functional impairment in daily life regarding sleep and rest. Patients are instructed to check those items that are applicable to them. Higher scores indicate more limitations in sleep and rest (range 0-499).

Physical activity was assessed in two ways. ***Objective physical activity*** was assessed with actigraphy. The actigraph is a motion-sensing device that gets attached to the ankle and was worn by patients for 12 consecutive days and nights an pre-and post-treatment. Twelve daily physical activity scores were calculated, expressed in the average number of accelerations per 5 minutes. An average daily level of physical activity was computed over this period with higher scores indicating more physical activity. Previous research supports the reliability and validity of the actigraph (7).
Second, ***self-reported perceived activity*** was assessed with the Checklist Individual Strength, subscale activity (8). Three items (e.g. ‘I don’t do much during the day’) assess patients’ perceived problems with their daily activity over the past 2 weeks. Items are scored on a 7-point Likert scale. Higher scores indicate more perceived problems with activity (range 3-21). In the Gielissen-trial, a previous version of the CIS-activity has been administered which differs from the recent version in the wording of the three items: the scoring is identical. Therefore, this was taken into analyses.

Problems with social support were assessed with the Sonderen Social Support Inventory (SSL) (9, 10). To asses a patients’ ***discrepancy between the amount of received and the amount of desired social support*** the subscale discrepancies is used (SSLD). Eight items (e.g. ‘What is your opinion about the extent to which people: Stand by you?’) with items scored on a 4-point Likert scale ranging from (1) ‘I miss it’ to (4) ‘It happens too often’. Higher scores indicate a higher discrepancy in support (range 8-32). To assess the patients’ ***perceived social support*** the subscale interaction is used (SSLI). Twelve items (e.g. ‘Does it ever happen that people give you compliments?’). Items score on a 4-point Likert scale ranging from (1) ‘rarely or never’ tot (4) ’very often’. Higher scores indicates higher social interaction. To assess the patients’ ***perceived negative interaction*** the subscale negative interaction is used. Social support negative interaction (SSLN).. 4-point Likert scale ranging from (1) ‘rarely or never’ tot (4) ’very often’. 7 items (range 7-28). Only measured at baseline in the Poort trial.

***Anxiety*** were assessed with the anxiety subscale of the Hospital Anxiety and Depression Scale (HADS). (11)Seven items (e.g. ‘I have been feeling tense lately’) assess patients’ anxiety level especially during the previous month. Items score a 4-point Likert scale, with differing responses per items (e.g. (3) ‘mostly’, (2) ‘often’, (1) ‘sometimes’ and (0) ‘not at all’. Higher scores indicate more anxiety (range 0-21). Anxiety was only measured at baseline.

***Depressive symptoms*** were assessed with the Beck Depression Inventory for Primary Care (BDI-PC) (12). The questionnaire consist of seven items (e.g. ‘Sadnesss’) with a 4-point Likert scale, with differing responses per item (e.g. (0) ‘I don’t feel sad at all’, (2) ‘I feel sad a lot of the time’, (3) ‘I feel sad all the time’, (4) ‘I am so sad that I cannot bear it’). Higher scores indicate higher levels of depressive symptoms (range 0-21). Depressive symptoms was only measured at baseline.

**Figure S1.** Moderation analysis.


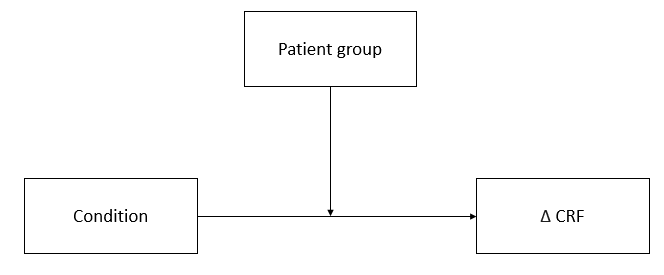


*Note: ∆ = residual change score
CRF = cancer-related fatigue*

**Figure S2.** Moderated mediation analysis.


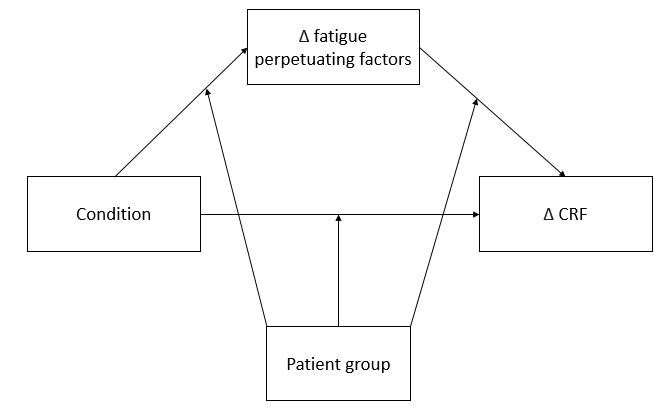


*Note: ∆ = residual change score
CRF = cancer-related fatigue*

**Table S2.** moderation analysis of the residual change of cancer-related fatigue by condition and patient group

|  | **B** | **Standard Error** | **p-value** |
| --- | --- | --- | --- |
| **Condition** | -1.05 | 0.11 | **<0.001*** |
| Patient group | -0.14 | 0.18 | 0.42 |
| **Condition * Patient group** | 0.51 | 0.22 | **0.022*** |
| Sex (covariate) | -0.04 | 1.06 | 0.74 |
| Age (covariate) | 0.01 | 0.01 | 0.27 |

*Note: *Significant at p<0.05 level.*

**Table S3.** Post hoc moderation analysis of the residual change of cancer-related fatigue by condition and patient group, including self-efficacy as covariate.

|  | **B** | **Standard Error** | **p-value** |
| --- | --- | --- | --- |
| **Condition** | -1.03 | 0.11 | **<0.001*** |
| Patient group | -0.73 | 0.18 | 0.68 |
| Condition * Patient group | 0.42 | 0.22 | 0.06 |
| Sex (covariate) | -0.03 | 1.05 | 0.75 |
| Age (covariate) | 0.004 | 0.01 | 0.50 |
| **Self-efficacy (covariate)** | -0.05 | 0.02 | **<0.001*** |

*Note: *Significant at p<0.05 level.*

**Table S4.** Post hoc moderation analysis of the residual change of cancer-related fatigue by condition and patient group, including depressive symptoms as covariate.

|  | **B** | **Standard Error** | **p-value** |
| --- | --- | --- | --- |
| **Condition** | -.99 | 0.15 | **<0.001*** |
| Patient group | -0.12 | 0.20 | 0.55 |
| Condition * Patient group | 0.45 | 0.24 | 0.06 |
| Sex (covariate) | -0.20 | 0.19 | 0.28 |
| Age (covariate) | 0.004 | 0.01 | 0.63 |
| **Depressive symptoms (covariate)** | 0.05 | 0.02 | **0.02** |

*Note: *Significant at p<0.05 level.*

**Figure S3.** Comparison with T3 of the Poort trial.


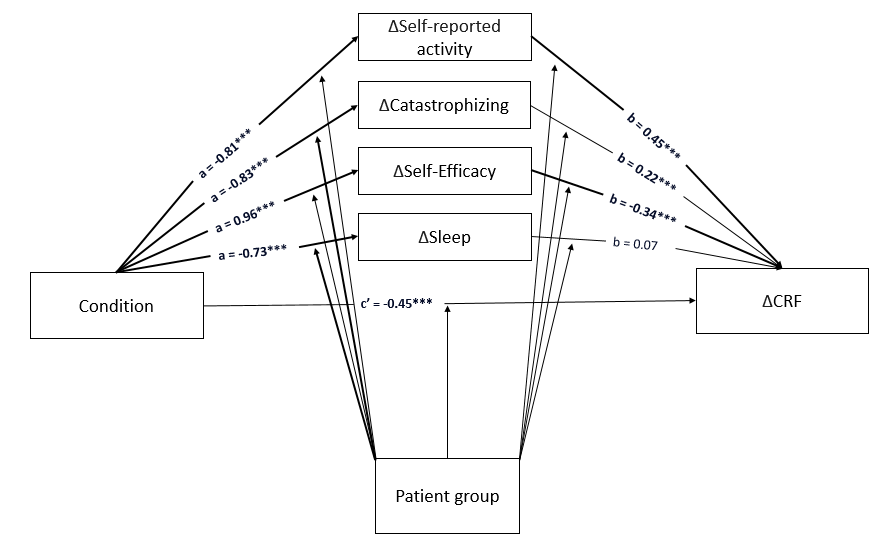


*Note: *p<0.05, **p<0.01, ***p<.001
∆ = residual change score*

*CRF = cancer-related fatigue*

**References**

1. Creamer M, Bell R, Failla S. Psychometric properties of the Impact of Event Scale - Revised. Behav Res Ther. 2003;41(12):1489-96.

2. Horowitz M, Wilner N, Alvarez W. Impact of Event Scale: a measure of subjective stress. Psychosom Med. 1979;41(3):209-18.

3. Ray C, Weir W, Stewart D, Miller P, Hyde G. Ways of coping with chronic fatigue syndrome: development of an illness management questionnaire. Soc Sci Med. 1993;37(3):385-91.

4. Jacobsen PB, Andrykowski MA, Thors CL. Relationship of catastrophizing to fatigue among women receiving treatment for breast cancer. J Consult Clin Psychol. 2004;72(2):355-61.

5. Prins JB, Bleijenberg G, Bazelmans E, Elving LD, de Boo TM, Severens JL, et al. Cognitive behaviour therapy for chronic fatigue syndrome: a multicentre randomised controlled trial. Lancet. 2001;357(9259):841-7.

6. Bergner M, Bobbitt RA, Carter WB, Gilson BS. The Sickness Impact Profile: development and final revision of a health status measure. Med Care. 1981;19(8):787-805.

7. van der Werf SP, Prins JB, Vercoulen JH, van der Meer JW, Bleijenberg G. Identifying physical activity patterns in chronic fatigue syndrome using actigraphic assessment. J Psychosom Res. 2000;49(5):373-9.

8. Worm-Smeitink M, Gielissen M, Bloot L, van Laarhoven HWM, van Engelen BGM, van Riel P, et al. The assessment of fatigue: Psychometric qualities and norms for the Checklist individual strength. J Psychosom Res. 2017;98:40-6.

9. Timmerman I, Emanuels-Zuurveen E, Emmelkamp P. The Social Support Inventory (SSI): A Brief Scale to Assess Perceived Adequacy of Social Support. Clinical Psychology & Psychotherapy. 2000;7:401-10.

10. Sonderen E, Ormel J. Het meten van aspecten van sociale steun en hun relatie met welbevinden. Een onderzoek naar de bruikbaarheid van de SSL-I ende SSL-D. Tijdschrift voor Psychologie en Gezondheid. 1997;25.

11. Zigmond AS, Snaith RP. The hospital anxiety and depression scale. Acta Psychiatr Scand. 1983;67(6):361-70.

12. Beck AT, Guth D, Steer RA, Ball R. Screening for major depression disorders in medical inpatients with the Beck Depression Inventory for Primary Care. Behav Res Ther. 1997;35(8):785-91.
